# Photo-modulated optical and electrical properties of graphene

**DOI:** 10.1515/nanoph-2021-0582

**Published:** 2022-01-14

**Authors:** Hongyu Tang, Sergey G. Menabde, Tarique Anwar, Junhyung Kim, Min Seok Jang, Giulia Tagliabue

**Affiliations:** Laboratory of Nanoscience for Energy Technologies (LNET), École Polytechnique Fédérale de Lausanne (EPFL), Station 9, CH-1015, Lausanne, Switzerland; School of Electrical Engineering, Korea Advanced Institute of Science and Technology, Daejeon, Korea

**Keywords:** all-optical modulation, electrical properties, graphene, nanophotonic engineering, optical properties, photogating

## Abstract

Photo-modulation is a promising strategy for contactless and ultrafast control of optical and electrical properties of photoactive materials. Graphene is an attractive candidate material for photo-modulation due to its extraordinary physical properties and its relevance to a wide range of devices, from photodetectors to energy converters. In this review, we survey different strategies for photo-modulation of electrical and optical properties of graphene, including photogating, generation of hot carriers, and thermo-optical effects. We briefly discuss the role of nanophotonic strategies to maximize these effects and highlight promising fields for application of these techniques.

## 1 Introduction

Graphene is a two-dimensional (2D) hexagonal honeycomb lattice of carbon atoms. This atomic structure results in a lightweight material with exceptional electrical [1], optical [2], mechanical [3], thermal [4], and chemical [5] properties that have been exploited both for fundamental studies and device applications. In particular, graphene’s unique optoelectronic properties, including zero bandgap, high carrier mobility, electron–hole symmetry, and highly tunable interband and intraband optical transitions over a wide spectral range from visible to terahertz frequencies, offer unparalleled opportunities for signal transmission and modulation as well as light detection and emission [6, 7]. Leveraging these exotic optical and electronic properties along with a high thermal conductivity and exceptional mechanical properties, graphene has thus shown enormous potential in functional optoelectronic devices such as photodetectors [8], light-emitting diodes [9], optical-logic devices [10], and solar cells [11]. Furthermore, due to the nature of the π-bonding in graphene, electrostatic interactions at a doped-graphene/electrolyte interface have recently opened unexpected pathways for realizing electro-kinetic energy conversion devices [12] and neuromorphic computing systems [13].

Remarkably, contrary to conventional metals, the free carrier concentration and the associated Fermi level in graphene can be largely modulated via electrostatic gating or chemical doping, owing to its low intrinsic carrier density, low density of states, and 2D geometry [14]. Hence, the electrical and optical properties of graphene, which strongly depend on the Fermi level position, can be altered accordingly. This opens an unprecedented opportunity for realizing tunable devices.

Photo-modulation of the optoelectronic response of graphene has recently attracted increasing attention thanks to the possibility of achieving contactless reconfigurability and accessing an ultrafast modulation regime. In particular, leveraging state-of-the-art nanophotonic engineering solutions, such as plasmons [15], nano-antennas [16], and metasurfaces [17, 18], the photo-modulation of graphene can be controlled in a wide wavelength range. Photo-modulation would indeed be extremely advantageous for applications in the middle, far-infrared, and terahertz ranges where graphene plasmons can enable advanced photodetection, optical communication, and far-field light modulation [19], [20], [21]. Also, photo-modulation could enhance electro-kinetic effects, boosting the performance of energy conversion devices. Yet, a number of open fundamental and engineering problems still need to be addressed to fulfill the potential of graphene photo-modulation.

In this Review, we first give an overview of the fundamental electrical and optical properties of graphene. In particular, we focus on how graphene optoelectronic properties originate from its unique band structure and clarify how doping can modulate them. Subsequently, in the second part of the Review, we address fundamental processes that result in photo-modulation of the electrical and optical properties in graphene. Finally, in the third part of the Review, we present applications of all-optical modulation of graphene ranging from optoelectronic to energy devices. In particular, we discuss fundamental open questions and nanophotonic engineering approaches that can be leveraged to advance the performance and functionalities of these unique systems.

## 2 Fundamentals

The understanding of the physics of graphene has grown quickly over the years, leading to the identification of rich fundamental phenomena and the development of advanced devices. Here we focus on the key electrical and optical properties that are critical for understanding or subject to photo-modulation.

### 2.1 Electrical properties

**Figure 1:**
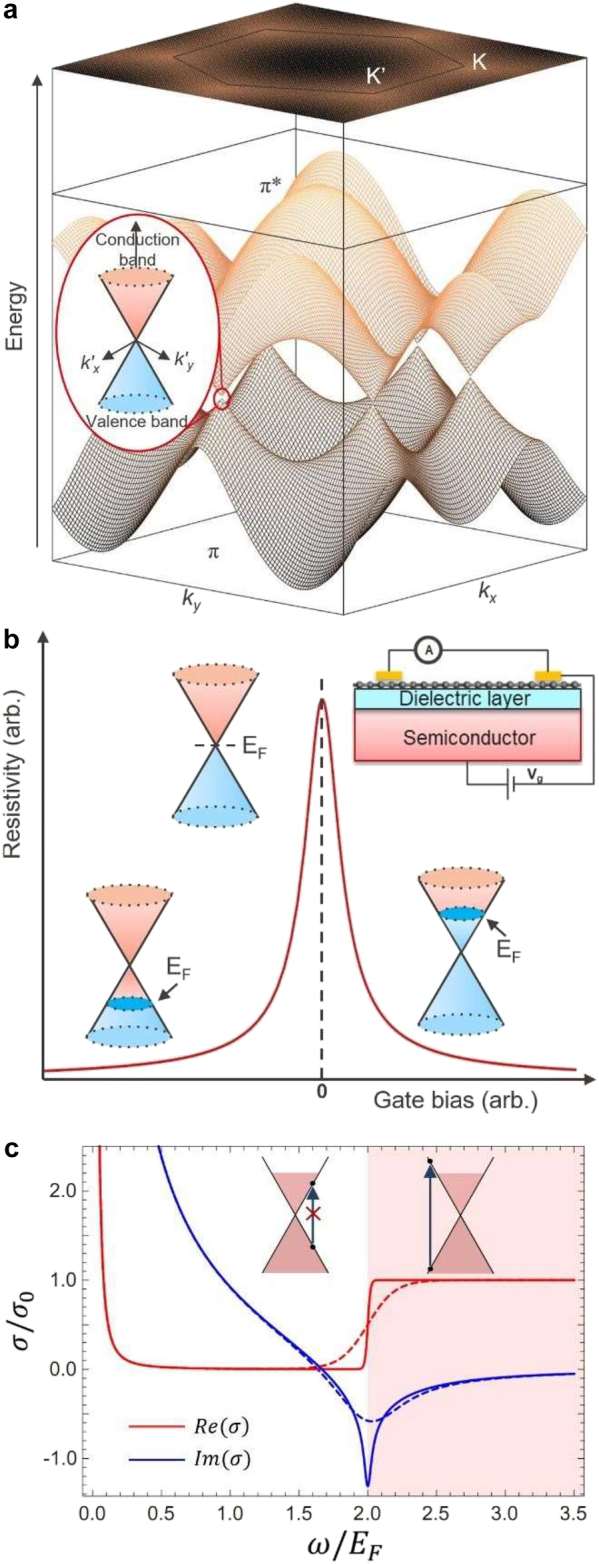
Electrical and optical properties of graphene. (a) Band structure of graphene. The top and bottom energy surfaces represent the conduction band (π* band) and the valence band (π band), respectively. Inset is the enlargement of the band structure near the Dirac point Κ_0_, showing the linear dispersion. (b) Resistivity of single-layer graphene as a function of an external gate bias showing the ambipolar electric field effect. Adapted with permission from [6]. (c) Optical conductivity of graphene, in units $\sigma_{0}=e^{2}/4\hbar$, as a function of frequency ω. The real part (Re(σ), red) and the imaginary part (Im(σ), blue) are plotted for low ($T/E_{\text{F}}=0.005$, solid), and high ($T/E_{\text{F}}=0.05$, dashed) temperature. The red shaded area denotes the regime of interband excitations. The inset schematic diagram explains the mechanism of Pauli blocking of photon absorption in graphene.

Graphene is a 2D planar crystal composed of a single layer of carbon atoms forming hybridized SP^2^ bonding in a hexagonal honeycomb lattice. Its single-layer thickness is only 0.34 nm and the C–C bond length *a* ∼ 0.142 nm. The electronic band structure of graphene can be described by using the tight-binding Hamiltonian [6, 7] and, within this π-band approximation, the dispersion relation of graphene can be expressed as:

(1) $$E^{\pm }\left(k_{x},k_{y}\right)=\pm \gamma_{0}\sqrt{3+2\text{cos}\left(\sqrt{3}k_{y}a\right)+4\text{cos}\left(\frac{\sqrt{3}}{2}k_{y}a\right)\text{cos}\left(\frac{3}{2}k_{x}a\right)}$$

where $\gamma_{0}$ ≈ 2.8 eV is a nearest neighbor hopping energy, which is the transfer integral between first-neighbor π-orbitals [7]. Here, the plus sign represents the conduction band (π* band) and the minus sign represents the valence band (π band). The band structure of graphene is shown in Figure 1a. In the 2D Brillouin zone near the *E*(*k*) = 0 points, called Dirac points, the π- and π*-bands are symmetrical to each other and both satisfy a linear dispersion relation *E* = ℏ*v*_F_*k*, where ℏ is the reduced Planck’s constant, and *v*_F_ ∼ 10^6^ m/s is the Fermi velocity in graphene. Thanks to its unique band structure, graphene exhibits an exceptionally high charge carrier mobility. Indeed, in suspended graphene at room temperature, the mobility can reach ∼200,000 cm^2^ V^−1^ s^−1^ [22], which is at least several times higher than that of traditional semiconductors (1–70,000 cm^2^ V^−1^ s^−1^) [23, p. 3]. The electronic transport properties can deteriorate by extrinsic scattering mechanisms involving charged impurities, surface roughness, or phonons in substrate materials [24]. Therefore, for device applications, controlling the quality of the substrate and interfaces is critical to ensure a good charge carrier mobility.

Importantly, thanks to the 2D nature and low density of states, the Fermi level of graphene can be efficiently modulated by applying an external electrostatic gating [6]. For intrinsic graphene, the Fermi level is at the Dirac point, which is typically set as the zero-energy reference. In this case, the carriers involved in conduction are both electrons and holes, which are called *bipolar* conductivity. As shown in Figure 1b, by applying a positive or negative gate voltage, the type and concentration of carriers in graphene can be continuously tuned between electrons and holes: a positive (negative) gate voltage makes the *E*_F_ higher (lower) than the Dirac point, which induces electrons (holes) with concentration *n* = *αV*_g_, where α=ϵ/d is the specific capacitance of the system, and ϵ and *d* are the dielectric constant and the thickness of the gate dielectric, respectively [25]. Carrier mobility is a weak function of the Fermi level, unless close to the Dirac point [6], the sheet resistance of graphene, *ρ*, is strongly affected by the carrier concentration. Thus, depending on the Fermi-level, *ρ* can vary from several thousand to several hundred Ω/sq. We note that, in addition to electrostatic gating, adsorption of impurities from the surrounding environment (e.g., water, oxygen) or charges in the supporting materials can also change the Fermi level of graphene. For example, adsorption typically induces p-type doping, leading to a negative Fermi level at zero gate bias [26].

The linear electronic dispersion of graphene also leads to a low electronic heat capacity $C_{\text{el}}=\alpha T_{\text{e}}$, where *T*_e_ is the electronic temperature of graphene, $\alpha =\left[\left(2\pi E_{\text{F}}\right)/\left(3\hbar^{2}v_{\text{F}}^{2}\right)\right]k_{\text{B}}^{2}$, and $k_{\text{B}}$ is Boltzmann’s constant [27]. When the electrons in graphene absorb energy from incoming light, its electronic temperature rapidly increases due to the low electronic heat capacity, causing a dramatic change in the optical and electronic properties of graphene. The photo-modulated electronic distribution can be found by determining the electronic temperature and the chemical potential of graphene while enforcing the conservation of electron population for a given optical fluence [28].

### 2.2 Optical properties

The exotic electronic band structure of graphene implies that its interaction with light is also unique. The optical conductivity of graphene, σ, strongly depends on the incident photon energy (ℏω) as well as the chemical potential (*μ*), the electronic temperature (*T*_e_) and the carrier scattering rate (Γ), the latter being often dominated by impurity scattering [29, 30]. We note here that in steady state, the electronic temperature is equal to the lattice temperature *T*. However, as we will discuss in the next section, at ultra-fast timescales the two differ. The optical conductivity can be decomposed into two separate contributions, associated with intraband and interband charge carrier excitations. The respective analytical expressions, derived by Falkovsky [31], are:

(2) $$\sigma^{\text{intra}}\left(\omega \right)=\frac{2e^{2}k_{\text{B}}T}{\pi \hbar \left(\Gamma -\text{i}\omega \right)}\text{ln}\left[2\text{ cosh }\left(\frac{\mu }{2k_{\text{B}}T}\right)\right]$$

(3a) $$\sigma^{\text{inter}}\left(\omega \right)=\frac{e^{2}}{4\hbar }\left[G\left(\frac{\omega }{2}\right)-\frac{4\omega }{\text{iπ}}\int_{0}^{\infty }\frac{\left(G\left(E\right)-G\left(\frac{\omega }{2}\right)\right)}{\omega^{2}-4E^{2}}\text{d}E\right]$$

(3b) $$G\left(E\right)=\frac{\text{sinh}\left(\frac{E}{k_{\text{B}}T}\right)}{\text{cosh}\left(\frac{\mu }{k_{\text{B}}T}\right)+\text{cosh}\left(\frac{E}{k_{\text{B}}T}\right)}$$

The overall optical conductivity of graphene as a function of the incident photon energy (ℏω) is shown in Figure 1c. One immediately notices that the real part of the conductivity (red curve) shows a step at ω = 2*E*_F_, which corresponds to the onset of the interband transitions. For ω < 2*E*_F_, indeed, interband transitions are forbidden due to Pauli blocking, schematically illustrated in the left inset of Figure 1c. Here, the optical conductivity of graphene is determined mainly by intraband transition, which can be reduced to a Drude form in the low temperature limit (*k*_B_*T* ≪ *E*_F_) [31]. In this regime, which typically spans from THz to IR depending on the Fermi level, the optical properties of graphene are highly sensitive to the doping level that can be controlled electrostatically, chemically or optically, as we will discuss in the next section. On the other hand, when ω > 2|*E*_F_| (red shaded area in Figure 1c), interband transitions dominate the optical response of graphene. Due to its linear band structure, at steady state, graphene exhibits a constant sheet conductivity $\sigma_{0}=e^{2}/4\hbar$, or equivalently, a spectrally flat absorbance $A=\pi e^{2}/\hbar c\approx 2.3\%$. Yet, as we will see in the next section, modulation of the optical conductivity in this regime becomes possible at ultra-fast timescales thanks to the generation and thermalization of a hot carrier (HC) population; this ultrafast tunability of graphene stems from the electronic temperature dependent Drude weight for the Dirac material [32].

The optical absorption by graphene can also depend on the intensity of incident light [33]. Under sufficiently strong optical excitation, the excited electron density in the conduction band becomes high enough to make further excitation of electrons increasingly inefficient. As a result, graphene is optically saturated and no longer absorbs light. This nonlinear optical behavior of graphene is quantified by the absorption coefficient as a function of light intensity as follows:

(4) $$\alpha \left(I\right)=\frac{\alpha_{\text{s}}}{1+I/I_{\text{S}}}+\alpha_{\text{NS}}$$

where *α* is the absorption coefficient, *I*_S_ is the saturation intensity and $\alpha_{\text{S}}$ and $\alpha_{\text{NS}}$ are the saturable and nonsaturable absorption components. However, due to graphene’s exceptional band structure, the experimental data for nonlinear absorption fits well with the inverse square root dependence, which essentially means that the high intensity decay is slower than other materials. Marini et al., have reported a semi-analytical approach of graphene saturable absorption [34]. By considering both interband and intraband contributions to nonlinear absorption they find a significant dependence of fermi energy, and the electronic temperature which becomes important under intense optical pumping. Compared to other materials such as single-walled carbon nanotubes (SWCNTs) [35] and semiconductor saturable absorber (SA) mirrors [36], graphene has an order of magnitude lower saturation intensity. Apart from the saturation intensity, two other key parameters for quantifying the performance are the modulation depth, which is the maximum possible change of optical loss, and the nonsaturable loss, which is the part of losses that cannot be saturated. Moreover, graphene has a much lower nonsaturable loss, which results in a 2–3 times higher modulation depth compared to the above-mentioned materials. This property can be utilized to generate ultrashort laser pulses, which will be discussed in Section 4.4.

### 2.3 Hot carrier dynamics

**Figure 2:**
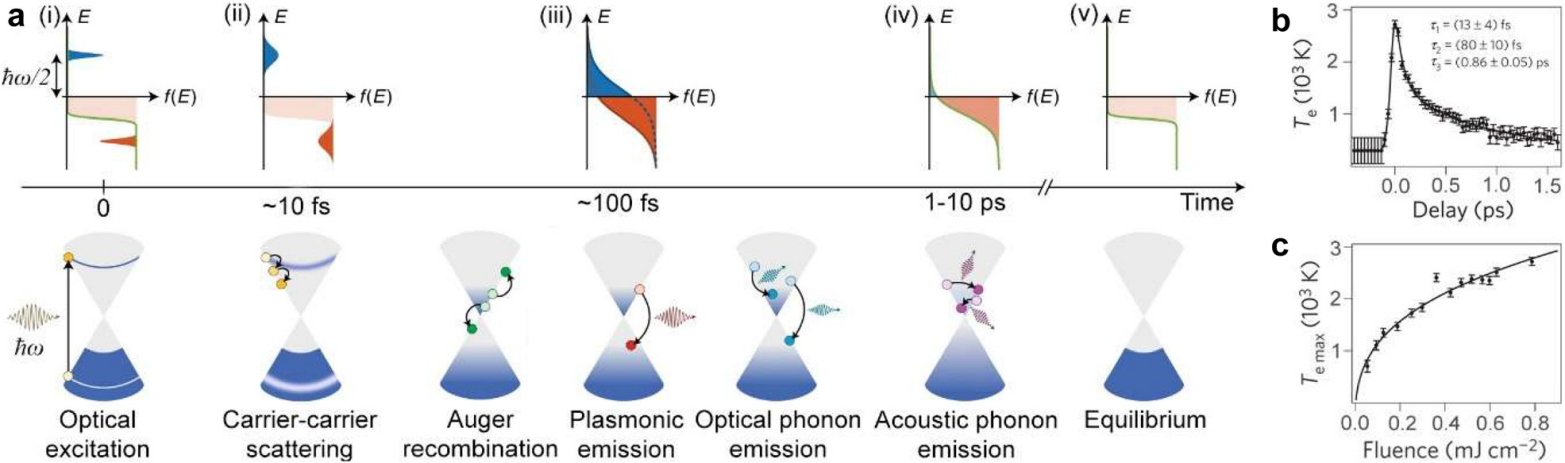
Hot carrier dynamics in graphene. (a) (i) Interband carrier transition upon optical excitation and the corresponding Fermi–Dirac distribution of carriers. (ii) Carrier–carrier scattering at a femtosecond timescale. (iii) A quasi-equilibrium state of carriers (electron and holes exhibit different temperatures). (iv) Interband thermalized carriers transition at a picosecond timescale, forming a single Fermi–Dirac distribution. (v) Thermal equilibrium of hot carriers with the lattice. (b) The variation of the electronic temperature with delay time. (c) The maximum electronic temperature as a function of fluence. Panel (a) adapted from [37], Panel (b) and (c) adapted from [38].

Absorption of ultra-short laser pulses triggers photoexcitation of charge carriers in graphene. These subsequently exhibit a complex thermalization dynamics that we will briefly describe here, while referring the interested reader to an in-depth recent review on the subject [39]. For sufficiently large photon energies, interband transitions around the K point create a population of high-energy charge carriers, which do not satisfy the Fermi–Dirac distribution (Figure 2a.i). Within a few tens of femtoseconds, Coulomb carrier–carrier scattering drives the energy redistribution and lead to the formation of a thermalized hot carrier population. Due to the low heat capacity of the electron gas in graphene, this thermalized Fermi–Dirac distribution is characterized by an extremely high electronic temperature, *T*_e_, up to a few thousands of Kelvins (Figure 2a.ii and iii) [40, 41]. Pump–probe and ARPES experiments have shown that this early thermalization dynamics is strongly influenced by the initial doping level as well as the intensity of irradiation [27, 42, 43]. Indeed, for nearly intrinsic graphene $\left(k_{\text{B}}T>E_{\text{F}}\right)$ and interband photoexcitation conditions, the electron and hole distributions can initially (at <130 fs timescale) thermalize to different temperatures ($T_{\text{e}}\neq T_{\text{h}}$, “inverted state”, Figure 2a.iii), and only subsequently relax to a single distribution ($T_{\text{e}}=T_{\text{h}}$, Figure 2a.iv). Also, Auger processes, including carrier multiplication and Auger recombination, are expected to play a dominant role at low fluences and high doping levels [27, 38, 41, 42]. Furthermore, on a few hundreds of fs timescale, both optical phonon emission [44] and, as shown recently, plasmon emission [37] contribute to the relaxation of the hot carrier population. Subsequently, coupling of optical phonons with acoustic phonons and, where applicable, hot carrier coupling with substrate phonons, determine the cooling dynamics over a few ps timescale (Figure 2a.iv). It is to be noted that the so-called hot phonon bottleneck, i.e., the re-absorption of hot optical phonons by electrons close to the Dirac point combined with reduced emission of acoustic phonons, can slow down the return to equilibrium, ultimately limiting the overall time response of the system [40, 44], [45], [46] (Figure 2a.v).

As evident from pump–probe experiments, the fs-to-ps photoexcitation and thermalization dynamics of charge carriers is directly associated with the ultrafast modulation of the optical response of graphene (i.e., its optical conductivity). In particular, for ω > 2|*E*_F_|, at sub-100 fs timescales, the nonthermalized charge carrier population hinders further photon absorption and results in an abrupt increase of transmittance. Instead, during the cooling process, absorption progressively increases while transmittance decreases (Figure 2b). The peak in transmittance modulation matches the maximum electronic temperature achieved in the system, which also depends on the excitation fluence (Figure 2c).

Overall, combining the unique tunability of the optical properties with excellent mechanical and thermal stability, as well as one of the highest known carrier mobility, graphene is an excellent material for high speed optical modulation. The modulation speed is ultimately limited by hot carrier dynamics (photocarrier generation and recombination), which is on the order of ps. Thus, in theory, graphene has the potential to achieve a modulation speed as high as 500 GHz.

## 3 Photo-modulation processes

**Figure 3:**
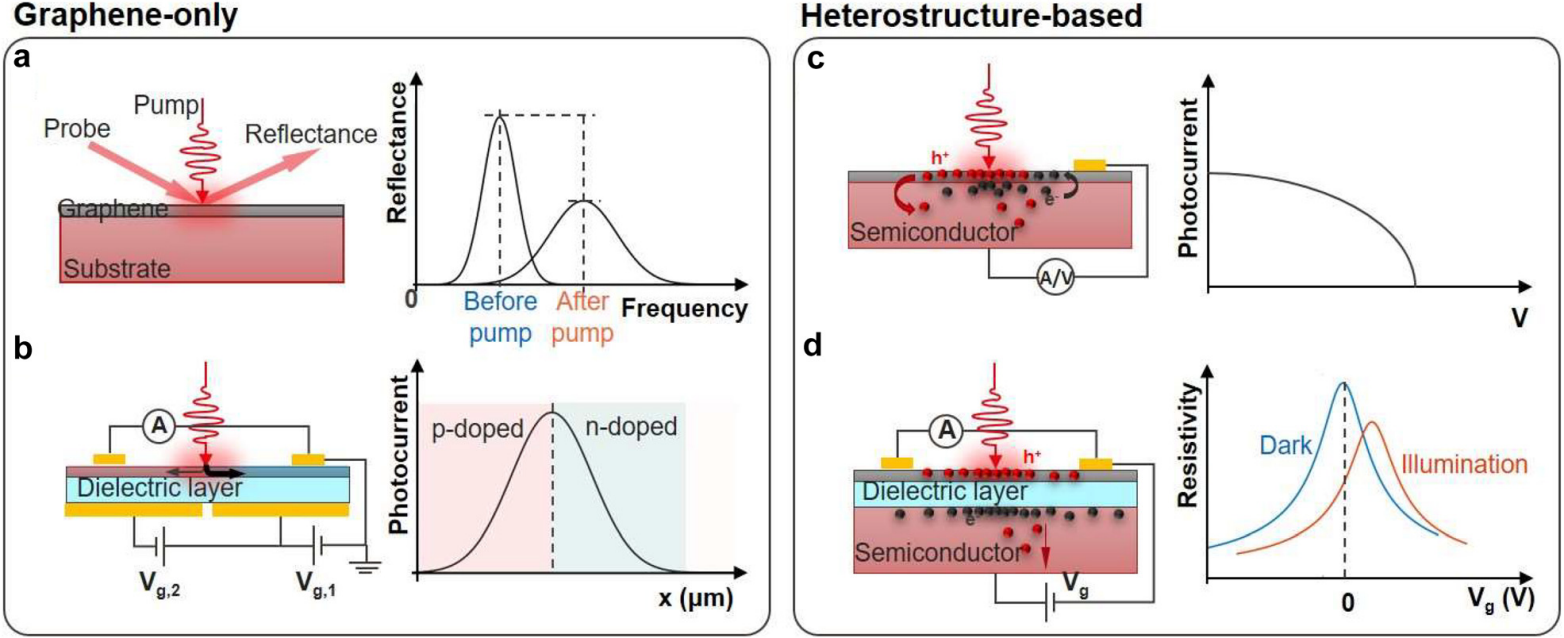
Overview of photo-modulation approaches. (a) Ultrafast optical modulation of graphene-only devices. The pump pulse generates a transient electronic response of graphene that in turns modulates the reflectance of the probe pulse. Controlling the time delay between the two pulses changes the modulation of the probe pulse. (b) Ultrafast electrical modulation of graphene p–n junction. Because the p-type and n-type graphene have different Seebeck coefficients, the absorbed light can increase the electronic temperature of graphene and further generate photocurrent due to photo-thermo-electric effects. The center of the illuminated region has the highest photocurrent. (c and d) Photo-modulation of graphene-based heterostructures. Two distinct ways can alter the graphene Fermi level and then change the photocurrent/resistivity via illumination: (c) by exchange of photo-generated charge carriers to/from an adjacent material, and (d) by the electrostatic screening of charges accumulated in a photoactive layer insulated from graphene via a dielectric layer, also called photo-gating.

As evident from the above discussion on fundamentals, the optical and electrical properties of graphene are strongly modulated by all phenomena that perturb its electronic states. In recent years, going beyond the electrical gating, a number of viable strategies for the photo-modulation of the electrical and optical properties of graphene have been identified. In particular, hot-carrier-driven effects have emerged as a promising solution for ultrafast modulation of the optoelectronic response of graphene. Also, coupling of graphene with photo-active materials, in particular 2D semiconductors, has opened new venues in the design of photogating architectures. In the following, we review the emerging photo-modulation mechanisms as well as associated device architectures and detection schemes (Figure 3). We first discuss the processes in which graphene is the only photoactive material (Section 3.1), and then review heterostructures-based approaches (Section 3.2).

### 3.1 Graphene-only processes

The ultrafast dynamics of the electronic and optical response of graphene can be leveraged in different ways to realize ultrafast optical and/or electrical modulation devices (Figure 3a and b). In the following sections we discuss two promising approaches that have recently emerged, namely the photo-thermo-optical (PTO) and the photo-thermo-electric (PTE) ones.

#### 3.1.1 Photo-thermo-optical (PTO) effect

Thanks to its metallic character in the mid-IR range, graphene supports surface plasmon polaritons (SPPs), which are collective oscillations of the electron cloud propagating along a metal/dielectric surface [15, 47]. SPPs uniquely enable extreme electric field confinement and enhancement at the nanoscale, leading to a strong light–matter interaction [48], [49], [50], [51]. Importantly, SPPs are highly sensitive to the optical properties of the metal as well as its dielectric surrounding [52]. Indeed, it was shown that electrostatic gating can be used to modulate surface plasmon modes in graphene [53] and, more recently, also in graphene–metal hybrid structures [16]. Based on our previous discussion of hot-carrier dynamics, it becomes evident that changes in optical conductivity due to pulsed irradiation can also be utilized to realize ultrafast modulation of surface plasmon modes in graphene in an all-optical manner. As these optical modulation approaches rely on the optical conductivity of graphene, $\sigma \left(\omega ,T_{\text{e}}\right)$, they are called *photo-thermo-optical* schemes. We will now discuss a few system design possibilities.

**Figure 4:**
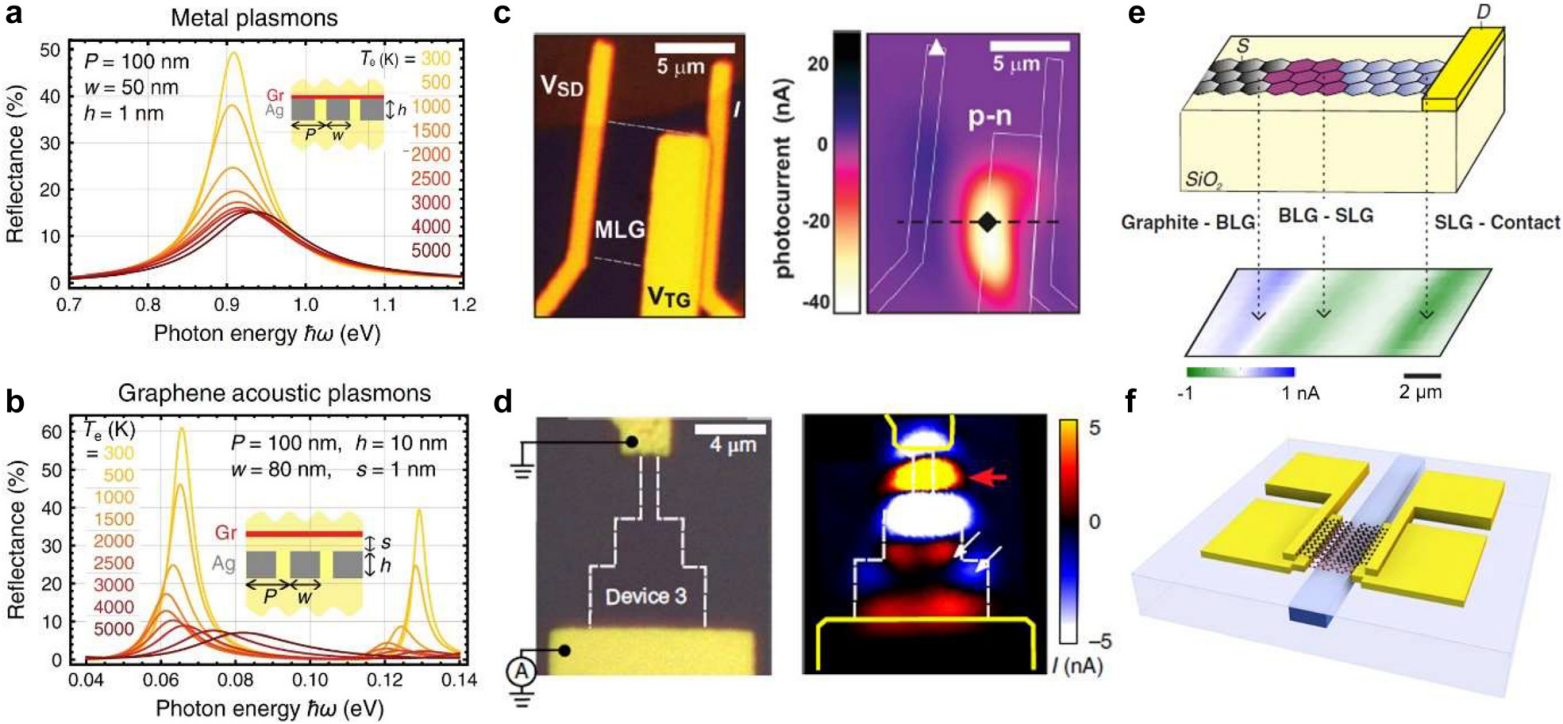
Photo-modulation processes of graphene: PTO and PTE. (a and b) PTO effect in graphene and graphene/metal hybrid systems. Variation in the reflection spectra of graphene/Ag system in the (a) NIR and (b) far/mid-IR plasmonic regions for different electronic temperatures, *T*_e_, in graphene, where *P*, *w*, *h* are geometrical parameters and *s* is the spacings. All dielectrics in the structure (yellow regions in the insets) are taken to have a permittivity *ε* = 2. (c–f) PTE effect in graphene-based devices. Panel (a) and (b) adapted from [54]. (c) Dual-gated device incorporating boron nitride top-gate dielectric and spatially resolved photocurrent map at *T* = 40 K with laser wavelength 850 nm. Adapted with permission from [60]. (d) Device with three rectangular graphene regions with increasing widths; the white arrows in the photocurrent map (right panel) indicate that the photocurrent is generated from the graphene edge, the red arrows denotes the photocurrent along the straight edges’ regions of single layer graphene. Adapted with permission from [61]. (e) Device layout and photocurrent map in the transparent substrate device, with a flake that contains adjacent regions of single- and bi-layer graphene, as well as graphene–metal contact. Adapted with permission from [62]. (f) Waveguide integrated PTE graphene photodetector with a split-gate geometry where PTE current is generated by absorbing the waveguiding modes. Adapted with permission from [63].

Direct excitation of SPPs with free-space irradiation is forbidden because of a significant momentum mismatch. One strategy to achieve light coupling to SPPs in graphene is to place a metallic grating of appropriate periodicity in its close proximity. It has been recently suggested that excitation of graphene acoustic plasmons in a graphene/spacer/metallic-grating heterostructure would exhibit a very strong dependence on the electronic temperature in graphene, which can be controlled via a pump beam. In particular, the reflectivity of a probe beam would be strongly decreased for high electronic temperatures due to a spectral shift and significant broadening of the plasmon mode (Figure 4a) [54]. Similar tendency – a decrease in absorption with increasing pump fluence (i.e., increasing electronic temperature) – was also reported experimentally at terahertz frequencies in Ref. [55], where the nonlinear absorption in graphene plasmonic nanoresonators was investigated with a terahertz pump–terahertz probe technique (see the recent review article [56] to get an overview of terahertz nonlinear optical properties of graphene).

In order to go beyond the mid-IR range and achieve visible-light all-optical modulation, it has been also suggested to use the sensitivity of surface plasmon modes in metallic nanostructures to the dielectric properties of the surrounding environment. Hence, in a graphene/metallic-grating heterostructure it is the metal plasmon resonances that can be tuned when a pulsed illumination increases the *T*_e_ in graphene and changes its σ[54]. However, in bulk metals the lossy nature of the plasmons significantly decreases the ensuing reflectivity modulation. Yet, the authors propose that in nm-thick metals large all-optical modulation would be possible (Figure 4b). Indeed, for a 1 nm thick silver grating, the predicted value of reflection modulation depth is as high as ∼70%.

To directly overcome the momentum mismatch problem, it has been recently proposed that structured light illumination could be utilized to create an “optically imprinted” grating on a graphene sheet. In fact, this would realize a spatial modulation of the local electronic temperature $T_{\text{e}}\left(x\right)$ and hence of the optical conductivity, mimicking the effect of a nearby metallic grating. The ultrafast transient nature of this grating would then enable time-dependent coupling of a probe beam to the plasmon mode and thus a modulation of its reflected intensity [54]. Alternatively, it has been also proposed that graphene nanostructures, i.e., nanoislands, could serve as ideal platform to achieve thermo-optical switching with a single plasmon [57].

These examples suggest a large, yet unexplored, potential of PTO effects in graphene for all-optical ultrafast modulation. Furthermore, they highlight the importance of optimized nanophotonic design for enhancing the modulation depth in these deeply subwavelength systems. Recently, intriguing applications in highly efficient THz high-harmonic generation [58] and highly sensitive microwave bolometry [59] have further confirmed the broad possibility to leverage PTO effects towards ultrafast and better performing light detection and emission devices.

#### 3.1.2 Photo-thermoelectric (PTE) effect

As we discussed in Section 2.3, following photoexcitation, the thermalized electron population with temperature $T_{\text{e}}$ remains out of equilibrium with the lattice for a few picoseconds [64]. Given a spatially nonuniform irradiation and graphene properties, the local temperature difference with the rest of the lattice produces thermal and charge currents. The magnitude and direction of the electric current depends on the carrier type and density [60, 65] (Figure 4c–f). In the case of homogeneous graphene, the thermal current from the hot spot is uniform in all directions, thus the cumulative electric current is zero. If the heated area is not uniform and contains areas of graphene with different electronic properties (e.g., p–n junction, single-/bi-layer interface, or simply areas with different doping levels), the symmetry of the electronic system is broken, and the integral electric current is not zero [60, 65]. The generated PTE bias depends on the Seebeck coefficient S in each region, and is given by $V_{\text{PTE}}=\left(S_{2}-S_{1}\right)\text{d}T$, where d*T* is the electronic temperature difference between the heated area and the surrounding lattice. In stark contrast to the semiconductor-based photovoltaic detectors, PTE exhibits a flat responsivity across the broad excitation spectrum (at constant power), meaning that a higher photon energy gives a larger photovoltage.

The PTE voltage can be generated not only at the boundaries in graphene, but also at the contact between graphene and metal [62], as well as in areas where system symmetry is broken by graphene patterning [61] (Figure 4d and e). It is to be noted that, besides the PTE effect, thermalized hot carriers can be directly harvested via the photovoltaic (PV) effect at the graphene–metal contacts where the energy bands adjust between the two materials with different Fermi level, forming a potential barrier [66]. However, experimental data indicate that the photovoltaic (PV) contribution to the cumulative photocurrent is insignificant compared to the PTE effect [62].

The short lifetime of thermalized photocarriers presents a technological challenge for their efficient harvesting. Recent progress in nanofabrication allowed for creation of complex devices with 3D architecture where p–n junction in graphene can be created, and PTE current detected at the same time. For example, PTE-based waveguide-coupled photodetectors have demonstrated a superior bandwidth exceeding 100 Gbit/s [63] (Figure 4f). Under external bias, such photodetectors can operate at a wide range of regimes: PTE, PV, or bolometric, and often are coupled with plasmonic elements which enhance local electric fields [67].

### 3.2 Heterojunction-based processes

Due to the linear and gap-less electronic dispersion near the Dirac point, the Fermi level of pristine graphene can be easily shifted by external conditions. Most commonly, this is achieved using an electrostatic gate bias. Nonetheless, there exist also two distinct ways to alter the graphene Fermi level via illumination: by exchange of photo-generated charge carriers to/from an adjacent material, and by the electrostatic screening of charges accumulated in a photoactive layer insulated from graphene via a dielectric layer, also called photo-gating (Figure 3c and d). In the following subsections, we discuss these two mechanisms.

#### 3.2.1 Charge transfer from/to graphene

When graphene is in direct contact with a photoactive material, charge transfer from/to graphene can occur under illumination, altering its Fermi level. Importantly, the flow of photoexcited carriers between the two materials, and hence the resulting photo-doping, is controlled by the band alignment, the interfacial properties, and the considered timescale.

As we discussed above, the photoexcitation of graphene results in the generation of hot carriers with high energy and ∼ps lifetimes, which is within the range of 1–100 ps lifetime typical for the photoexcited hot carriers in bulk semiconductor materials [68], but shorter than the hot carrier lifetime of 10–1000 ps recently reported for 2D semiconductors [69]. At graphene/semiconductor interface, the presence of an interfacial energy barrier (Schottky barrier) can lead to the selective transfer harvesting of one type of hot carriers into the semiconductor. Importantly, in addition to the energy requirement, restrictive momentum matching conditions must be simultaneously satisfied to achieve hot carrier transfer to the acceptor material. In this case, graphene thus acts as a photosensitizer for the adjacent material. Overall, this process, called the photo-thermionic emission (PTI), results in a PV effect, including the generation of a photocurrent and the photo-doping of graphene. We note that most commonly in the literature this effect is indicated with the acronym “PTE”. However, to avoid confusions with the photo-thermoelectric effect described in Section 3.1.2 we refer to it as “PTI”.

**Figure 5:**
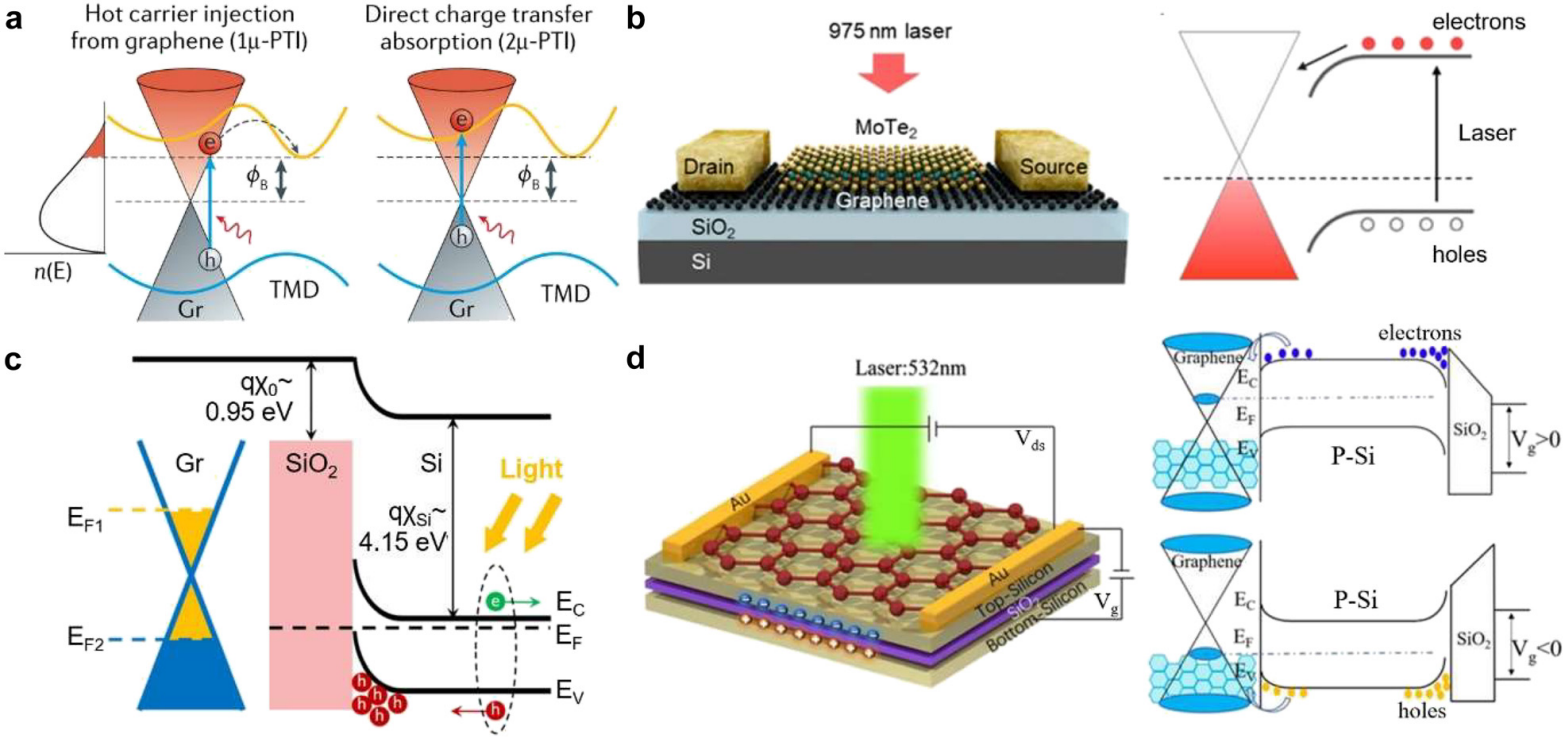
Photo-modulation processes of graphene-based heterojunctions. (a) Schematic representation of band diagrams for the two kinds of photo-thermionic charge transfer processes in a graphene/TMD heterostructure: (left) 1μ-PTI, (right) 2μ-PTI. The red shaded area is the distribution of ultrafast thermalized HCs, Φ_B_ is the Schottky barrier between graphene and TMDs [70]. (b) Graphene/MoTe_2_ photodevice where bands of MoTe_2_ bends upon the contact with graphene, creating a large-area 2D interface with facilitated electron injection to graphene. Adapted with permission from [89, p. 2]. (c) Energy band alignment of graphene on Si/SiO_2_ substrate. The band bending at the Si/SiO_2_ interface induces electron–hole pairs separation. Under the gate bias, electrons (green) diffuse into the Si bulk, while holes (red) are trapped at the Si/SiO_2_ interface. The accumulation of photo-generated holes at the interface acts as an additional gate bias, increasing the Fermi level in graphene and its n-type doping. Adapted with permission from [94]. (d) (Left) Schematic of the graphene/silicon-on-insulator heterojunction device in photoconductive mode. (Right) Energy band diagrams of the photodetector under positive (top) and negative (bottom) vertical voltage showing opposite sign of the photogating in graphene. Adapted with permission from [91].

While graphene hot electrons are generated near the K point, the conduction band minimum (CBM) of conventional bulk electron acceptors, such as TiO_2_, ZnO, etc., is at Γ point. Therefore, the large momentum mismatch hinders efficient collection [70, 71]. Luckily, advancements in low-dimensional Van der Waals (VdWs) materials have produced a whole new family of 2D semiconductors, including transition metal dichalcogenides (TMDs) that exhibit a CBM near K point, such as WSe_2_. These graphene/TMDs heterointerfaces have thus advanced the exploitation of the PTI pathway and the measurement of the associated photocurrent, which has a timescale of ∼ps (Figure 5a) [72]. Yet, the rapid thermalization of hot carriers and their inter-layer migration still limit the achievable internal quantum efficiency. We note that at picosecond timescales, the electron and hole distributions have already thermalized with each other and a single chemical potential, μ, is sufficient to describe the carrier distribution. Thus, this injection process is also called the 1μ-PTI pathway.

Graphene/TMDs heterointerfaces have also revealed a different injection pathway that occurs at much shorter timescales, and is named 2μ-PTI pathway (Figure 5a) due to the involvement of two distinct chemical potentials for electrons and holes, respectively. It relies on the photoexcitation of hot carriers directly across the interface, i.e., hot electron in graphene and hot hole in TMD, using photons with sub-TMD-bandgap energies. Several experimental results show that photocurrent generated through the 2μ-PTI pathway is ultrafast (∼fs) [71, 73]. Using transient absorption spectroscopy, He et al. indeed observed ultrafast rising time (∼27 fs) in the differential reflection signal, ascribed to this process. They also observed an ensuing prolonged decay (∼1.2 ps) which corresponds to the back transfer and recombination of hot carriers in graphene [71, 74]. For completeness, we note that hot carrier extraction from graphene can be also obtained in graphene/dielectric/metal heterostructures, where a VdWs dielectrics (e.g., boron nitride) is used as a potential barrier between graphene and the conductor [75, 76]. In particular, it has been shown that photo-induced tunneling across the VdWs heterostructure exhibits extremely low dark current owing to the large electronic barrier [77], [78], [79].

In addition to being used as a hot carrier injector, graphene can be employed as an efficient carrier acceptor in TMD-based heterostructures. For example, the recently reported WS_2_/graphene heterojunction exhibits ultrafast selective charge (hole) transfer into graphene [80]. In a MoS_2_/graphene system it has also been shown that the carrier extraction efficiencies from MoS_2_ are 93 and 81% for the A- and B-exciton, respectively. Additionally, the efficiency for the C-exciton, which uniquely originates from the band nesting region on the higher-energy side, is 80% [81]. It is important to note that the latter process exhibits a slow carrier relaxation (∼350 ps) that favors hot carrier harnessing and therefore energy conversion. Finally, we remark that in low dimensional heterostructures, complex charge and energy transfer (i.e., Dexter or Förster) processes can occur simultaneously [82]. Distinguishing and controlling their separated contributions will play an important role in advancing photodetection and energy conversion devices.

Charge carrier generation in a photoactive material can be used to control the doping of graphene also at much-slower timescales. In fact, depending on the band-alignment, either holes or electrons can be transferred to graphene, resulting in p-type or n-type doping, respectively. Among the numerous photo-active materials, metal halide perovskites have a band structure that is ideally fit for hole transfer to graphene. This makes graphene and its derivatives a popular choice for facilitating the hole transport in perovskite-based solar cells [83]. Therefore, from the photo-doping perspective, perovskite-based heterostructures can be optimized for the efficient hole doping of graphene. For example, graphene photo-doping up to 2.7 × 10^12^ cm^−2^ has been demonstrated in a heterostructure of graphene/MoO_3_/PEDOT:PSS/MAPbI_3_, which is optimized for the efficient hole transport [84]. Despite the high efficiency of carrier generation, instability under ambient conditions and fabrication difficulty still limit a wide employment of perovskite materials. On the other hand, the use of highly stable TMDs once again provides appealing opportunities in this direction. For example, a graphene/MoS_2_ system has been reported to provide an efficient electron transport to graphene due to the built-in potential formed at the interface, leading to a very high responsivity of 10^8^ A/W at room temperature [85, 86]. Alternative low-dimensional materials such as SWCNTs [87] and hexagonal boron nitride (h-BN) [88] have been shown to provide charge transfer to graphene as well.

Realization of tunable doping, both with 2D and bulk semiconductors, has recently opened new opportunities for graphene modulation. For example, it has been shown that the band bending at the graphene–MoTe_2_ interface depends on the input laser power and graphene Fermi level. Hence the selective transport of electrons or holes becomes possible by controlling these parameters (Figure 5b) [89, 90]. An analogous result has been reported also for a graphene/Si/SiO_2_/Si heterostructure, where the top Si layer, in contact with graphene, is gated by the bottom Si bulk, providing a tunable and fast carrier injection mechanism [91] (Figure 5d). In such a configuration, electron–hole pairs generated in the top Si are separated under the built-in potential at the heterojunction with graphene. By adjusting the carrier concentration in top Si, the increased built-in potential improves the separation efficiency of Si photocarriers. Furthermore, the carrier concentration gradient formed by the accumulation of carriers speeds up the diffusion of photogenerated carriers to the heterojunction region, leading to high gain and fast photoresponse.

Alternatively, by creating a wide-bandgap/graphene heterojunction, diffusion and transfer of both charge carriers can be obtained. This process, called diffusion pumping, can lead to a population inversion in graphene and could be leveraged to achieve terahertz emission for lasing or to efficiently modulate Dirac plasmons [92, 93].

Finally, we note here that advances in material synthesis have recently enabled the creation of controlled lateral heterojunctions instead of vertical ones [95]. This thus holds great promise for the exploration of novel hot carrier transfer and harnessing processes [96].

#### 3.2.2 Photo-induced electrostatic gating of graphene

In contrast to photo-doping by directly injected charge carriers, photo-gating of graphene is induced by electrostatic screening of charges accumulated in a photoactive material, which is not in direct contact with graphene. Effectively, the photo-active material plays a role of a photo-gate electrode that absorbs the incident light and generates photocarriers. Thus, the efficiency and feasibility of photo-gating solely depend on the photovoltaic properties of the active layer in the GFET-like device configuration, which alleviates band engineering constraints at the graphene interfaces (Figure 5c). Therefore, a wide variety of photodetectors have been demonstrated, covering a wide spectrum range. In the visible and near-infrared regimes, there is a broad choice of bulk semiconductor materials to be used as photo-gates which can provide high responsivity and on/off ratios exceeding 10^4^ (Figure 5c) [94, 97], [98], [99], [100]. For the mid-IR spectrum, pyroelectric photoactive materials such as LiNbO_3_ have been suggested for the role of the photo-active graphene substrate where photo-generated electron–hole pairs are redistributed in a way to create charged layer under graphene [101].

## 4 Applications

**Figure 6:**
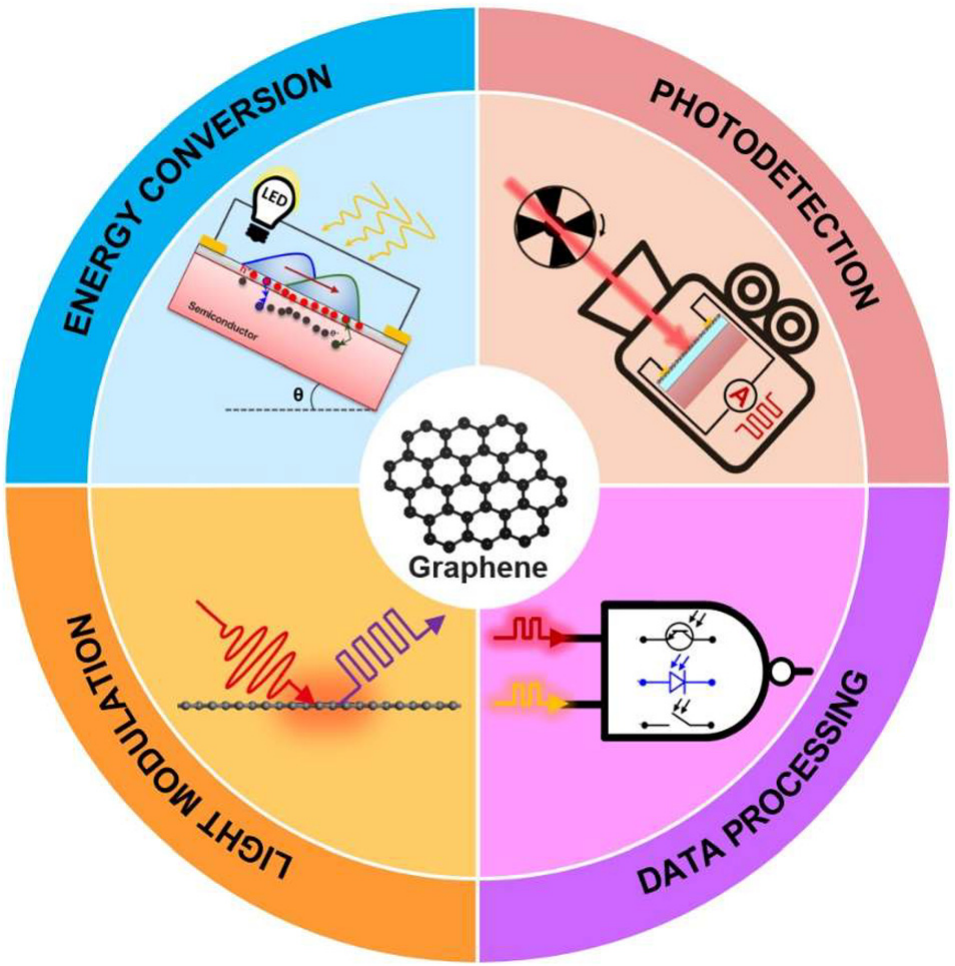
Schematic representation of the application domains for all-optical modulation of graphene.

In this section we discuss devices that use the photo-modulation mechanisms described in Section 3 as well as their application domains. In particular, we focus on photodetection, data processing, energy conversion and light modulation applications (Figure 6). In each section, we discuss state-of-the-art devices and nanophotonic engineering aspects that contributed to the improvement of the performance. Considering a common point of view for such different device functionalities, we hope to stimulate a cross-over of ideas that could spark further technical advancements and performance improvements.

### 4.1 Photodetection

Due to the unique physical properties discussed in previous chapters, graphene has been applied to a great variety of photodetection schemes. On the one hand, the saturable absorption (Section 2.2) and the ultrafast hot carrier dynamics (Section 2.3) in graphene potentially provide a platform for the ultra-broadband photodetection of light waves spanning all the way from mid-IR to visible. On the other hand, 2D nature of graphene significantly limits the responsivity of graphene-only photodetection schemes, and the same ultrafast carrier dynamics hinders the efficient harvesting of generated photo-carriers. Therefore, graphene has been typically used in combination with other materials in order to boost either the responsivity, or the photo-carriers extraction efficiency, or both. In particular, photo-modulation (via photo-doping or photo-gating) of the resistivity of graphene (electrical detection) under continuous-wave (CW) illumination serves as the basic photodetection mechanism. Given the vast literature already available on the topic, in this section, we summarize only the most recently reported state-of-the-art photodetectors demonstrating superior characteristics in the field, and we instead refer the interested reader to the following recent reviews on the subject [102], [103], [104].

**Figure 7:**
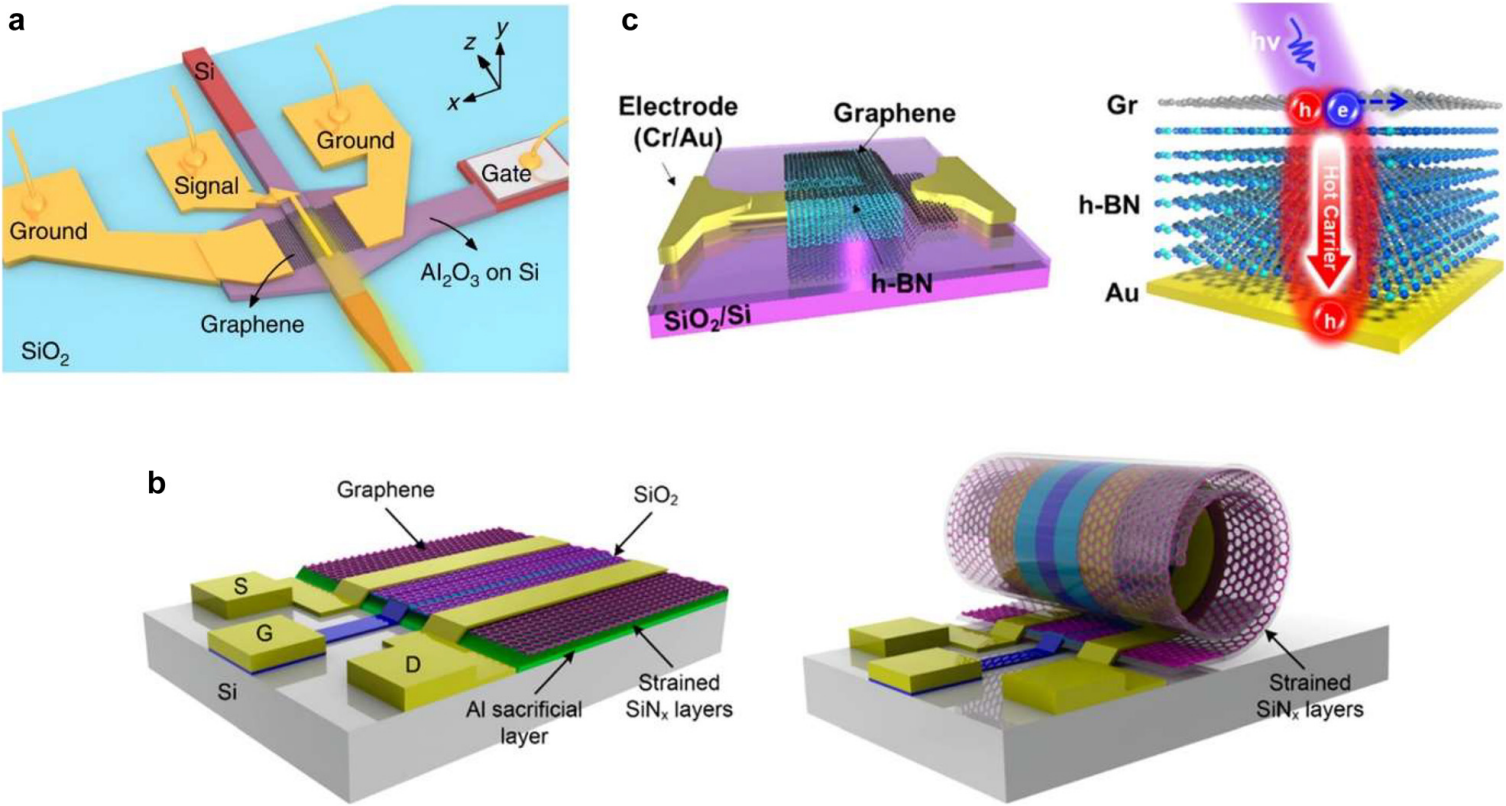
Photodetection applications of graphene. (a) Waveguide integrated graphene photodetector with a narrow plasmonic waveguide for facilitated light absorption in graphene. Adapted with permission from [67]. (b) 3D GFET device before (left) and after (right) roll-up process. 3D photonic nanocavity significantly enhances light–matter interaction. Adapted with permission from [110]. (c) Schematic of the graphene/h-BN/Au heterostructure photodetector (left) where photoexcited hole-type carriers transport through the h-BN layer either by tunneling or thermal distribution (thermionic) transport (right). Adapted with permission from [75].

One of the most prospective applications for graphene photodetectors is in waveguide-integrated devices operating at telecom frequencies. Indeed, the 2D nature of graphene perfectly suits the size limitations of waveguide-integrated optics. Recently, an ultra-high responsivity of 1.48 × 10^5^ A/W at 1550 nm wavelength (91.5 pW input power) was demonstrated by a device with carbon nanotubes (CNT) deposited on graphene [105]. In this device, photocurrent increased by 6.5 times compared to that without CNT, and the average response time was 83 ms. Another waveguide-integrated photodetector utilizes plasmonic elements to enhance the optical response in graphene by field localization [67] (Figure 7a). Reported responsivity at 1550 nm was 0.4 A/W, with operation bandwidth exceeding 40 GHz. While such responsivity value is the record-breaking among similar waveguide-integrated devices, the operation bandwidth reported for the alternative designs is higher, and exceeds 100 GHz [106, 107]; to put that responsivity and bandwidth numbers into perspective, similar values have been reported for the state-of-the-art semiconductor-based waveguide-integrated photodetectors [108]. As in other graphene–metal devices, PTE effect dominated under zero source-drain bias, while (much stronger) bolometric and photovoltaic effects dominated the photocurrent response when source-drain bias was applied.

Combination with plasmonic elements also led to a broadband photodetector operating in the visible spectrum where noble metals show a strong plasmonic response. In such device, the absorption of light is mostly due to the plasmonic response of the metallic metamaterial, while the photocurrent is generated by the injection of hot carriers from metal to graphene [109]. Reported responsivity is consistently above 140 mA/W for the whole visible spectrum from 450 to 700 nm wavelengths, while the absorption efficiency of the metamaterial stayed above 40%.

In order to increase the light absorption efficiency in the device, a rather exotic scheme has been proposed, based on a rolled multilayer structure with graphene, forming a 3D photoactive micro cavity [110] (Figure 7b). The rolling of the structure into a tubular graphene FET (GFET) increased the responsivity from 1.6 μA/W to 2.3 mA/W, measured at 325 nm wavelength under zero gate bias where the optical cavity resonance is excited. At the same time, responsivity exceeded 1 A/W when the gate bias was applied. Furthermore, the 3D GFET provides an ultra-broadband response covering the frequencies from UV to far-IR with responsivity mostly larger than 0.1 A/W, while the reported response time is < 0.3 ms. Needless to say, such a broadband photoresponse is impossible to obtain in semiconductors with finite and fixed bandgap. At the same time, responsivity of the recently reported avalanche photodetectors for visible light does not exceed 0.8 A/W with the typical operation bandwidth of ∼10 GHz [111], and the silicon-on-insulator-based photodetectors offer the responsivities less than 0.2 A/W at the optimal wavelength [112].

Another case of an ultra-high responsivity of 3.2 × 10^7^ A/W at visible spectrum (input power ∼10^−13^ W) was reported in a GFET device in which an organic semiconductor was deposited on graphene as the active layer [113]. This device also shows a fast response time of 1 ms. However, this device suffers from a very high dark current, since graphene is doped by the immediate contact with a photoactive material. To overcome this limitation, a device configuration with a thin gate dielectric has been proposed [75] (Figure 7c). In this case, photoresponse is induced by the tunneling of the photo-generated hot carriers from graphene to the underlying gate electrode. The proposed device uses a few-nm-thick hBN as the insulating layer, providing a high electron barrier of 2.27 eV at the graphene–hBN interface, while having a lower energy barrier of 1.93 eV at the hBN–gold interface. This device demonstrates a virtually zero dark current of ∼10^−13^ A and a few-microsecond-scale response time. The reported photo/dark current ratio is as large as 225 with 7 nm-thick hBN. We note that the 2D materials-based tunneling photodetectors is a new and rapidly growing field, where graphene often plays a key role to obtain the best responsivity values exceeding 10^3^ A/W [114].

The PTE, photovoltaic, and pyroelectric effects in graphene are successfully applied for the mid-IR photodetection, providing the similarly high responsivity of ∼10^3^ V/W as in the semiconductor-based devices, while having much faster response time [102]. This performance is due to the fast carrier dynamics and large Seebeck coefficient in graphene reaching 100 μV/K [115], which is comparable to 50–700 μV/K in the established thermoelectric materials such as Bi_2_Te_3_ and Sb_2_Te_3_ [116].

Graphene-based devices in recent years have demonstrated superior performance compared to the commercial products. In particular, they exhibit exceptionally broadband and ultrafast response as well as high responsivity values (Table 1). While nanophotonic engineering has already significantly contributed to the performance advancement of these devices, especially in terms of responsivity and bandwidth, there are still large margins of improvements for the speed of operation. In particular, based on our discussion is Section 2, it is clear that using hot carrier dynamics operation frequencies as high as 500 GHz could be possible. We would also like to highlight that, as noted by Kim et al. [84], the commonly used responsivity is not a good measure for the efficiency of carrier generation and transport in the devices with lateral 2D channels. The responsivity is defined as the ratio of the photocurrent to the incident light power. Unlike in vertical devices, where both the photocurrent and the incident light power can be normalized by the same active area, the responsivity of a lateral device strongly depends on the device geometry, which makes it difficult to compare the intrinsic performance among different devices.

**Table 1:** Performance comparison of commercial photodetectors and graphene-based prototypes.

Type		Responsivity	Response time	Spectral range	Operating temperature	Reference
Commercial products	Si	0.1–25 A/W	1–100 ns	200–1100 nm	Room T	Thorlabs
	InGaAs	∼1 A/W	∼10 ns	800–1700 nm	Room T	
	PbSe	∼3000 V/W	∼10 μs	1.5–4.8 μm	Room T	
	HgCdTe	∼2000 V/W	∼0.4 μs	2–24 μm	77 K	InfraRed Associates
Graphene-based prototypes	Hot carriers multiplication	1–10 A/W	∼20 ps	0.8–20 μm	Room T	[100]
	3D GFET	0.1–2.3 A/W	<0.3 ms	UV to THz	Room T	[110]
	Hot carriers tunneling	0.75 A/W	2.8 ms	Visible	Room T	[114]
	Plasmon-enhanced PV	∼0.4 A/W	<10 ps	1550 nm	Room T	[106]
	GFET heterostructure	3.2 × 10^7^ A/W	∼1 ms	Visible	Room T	[113]

### 4.2 Energy conversion

Graphene has a longstanding record of applications in energy conversion devices. Graphene and its derivatives have been explored as photosensitizers, transparent conductive electrodes, channels for charge transport, and catalysts in photoelectric and photoelectrochemical energy conversion devices. In this Review, we focus on application opportunities that leverage the tuning mechanisms described in Section 3. In particular, we discuss applications in photovoltaic devices as well as emerging iontronic systems, i.e., hydrovoltaic energy generators and ion pumps.

#### 4.2.1 Photovoltaic devices

**Figure 8:**
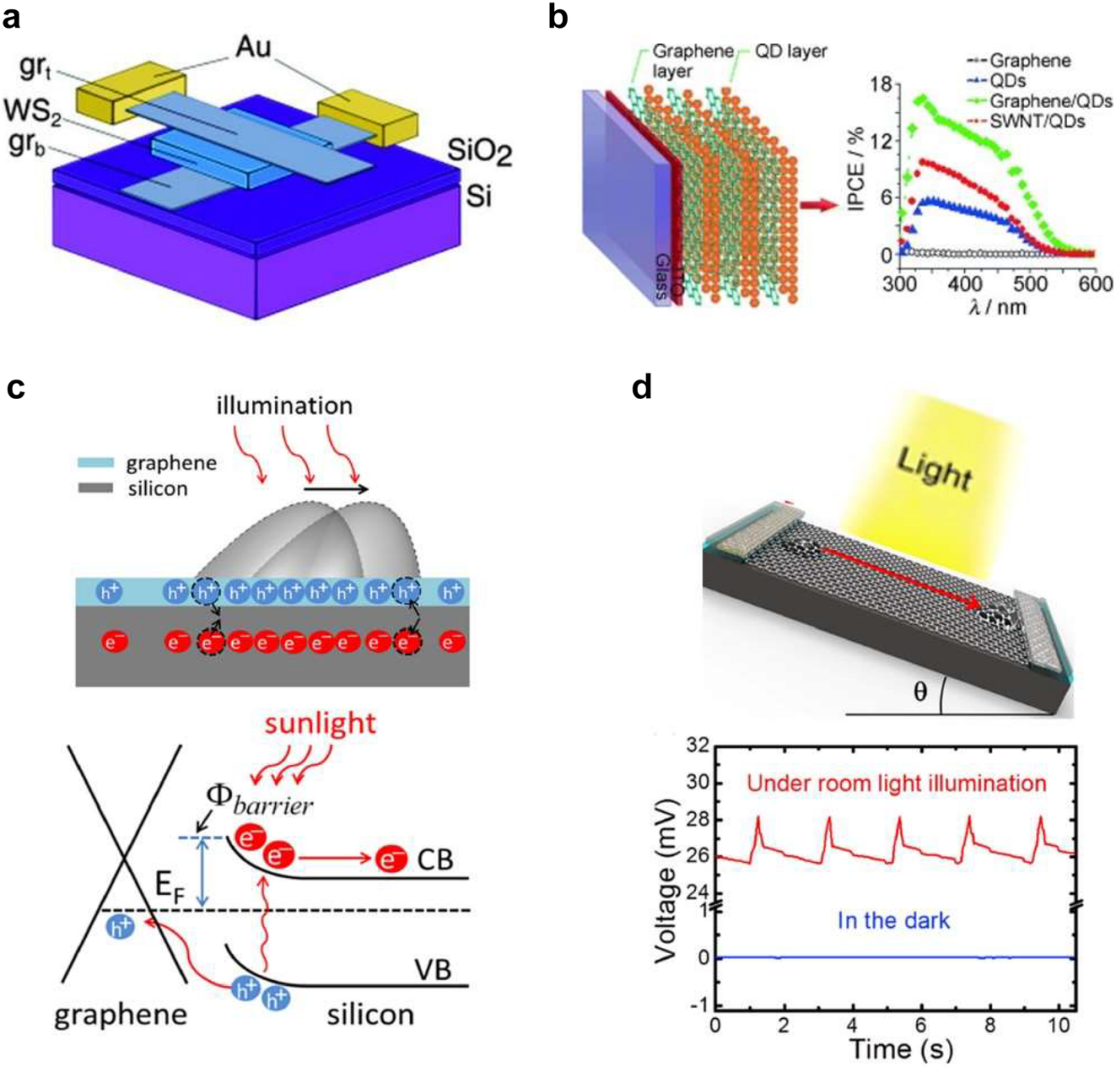
Energy conversion applications of graphene. (a) Graphene-TMDs vertical hybrid structure with graphene as transparent conductive electrode and WS_2_ as photosensitizer. (b) Graphene-based photovoltaic device with layered graphene and quantum dot (QD) films fabricated on transparent conducting indium tin oxide. (c) Top: Dynamic charge transfer when water is flowing over graphene arising from continuous doping and un-doping of the photoexcited carriers. Bottom: Schematic band profile of graphene–silicon nanogenerator. (d) A graphene-based nanogenerator harvesting light energy and enhanced voltage output under flow of water drops. Panel (a) adapted with permission from [119], Panel (b) adapted with permission from [123], Panel (c) and (d) adapted with permission from [12].

The high optical transmittance (∼97.7% [117]) and low sheet resistance of graphene make it suitable for use as a transparent conductive electrode (TCE) in solar cells, decreasing optical losses and enhancing the device performance. In particular, graphene TCEs have played a central role in the development of ultra-thin solar cells, entering the nm-thickness range, which will pave the way to ultra-light photovoltaic devices for portable and space application [118]. For instance, by creating a vertical hybrid structure with TMDs, in which graphene acts as a TCE, an external quantum efficiency greater than 30% is achievable [119] (Figure 8a).

Concurrently, broadband light absorption, high carrier-mobility as well as thermal and photochemical stability make graphene an attractive photosensitizer. The more detailed discussion (not just limited to photovoltaic devices) of charge transfer from/to graphene and how graphene acts a photosensitizer has been presented in Section 3.2 of the manuscript. We would like to highlight that graphene due to its broadband absorption gives rise to photogenerated electrons in conduction band of semiconductor, which is otherwise not possible by direct excitation due to wide band gap of semiconductor. Indeed, calculations for graphene-based organic PVs [120] show comparable efficiency to the state-of-the art organic PVs. Also, in dye sensitized solar cells (DSSCs), transition metals complexes and organic dyes are often used as sensitizers. Yet they suffer from expensive, time consuming preparation and narrow absorption-band with limited electrical conductivity. Thus, due to its superior and tunable optoelectronic properties, graphene can be an advantageous sensitizer both in organic PVs and DSSCs. Therefore different approaches have been utilized for using graphene as photosensitizers such as chemically functionalized graphene [121], graphene nanoribbons [120], and graphene-quantum dot hybrid systems [122, 123] (Figure 8b). Large improvements in the external quantum efficiency of graphene heterostructures for photovoltaic devices can also be achieved by nanophotonic engineering, in particular utilizing plasmonic nanoantennas. Indeed, it has been repeatedly shown that coupling of plasmonic nanostructures with graphene can increase light harvesting by one order of magnitude [51, 124].

Most remarkably, as discussed in Section 3.2, graphene/TMD heterojunctions pave the way to hot carrier photovoltaic devices, which promise to overcome the thermodynamic efficiency limit of conventional solar cells. Thus far, the efficiency of graphene-based solar cells governed by thermoelectric effects have been limited by low photovoltage, the photoresponse can be significantly improved where PV effect is dominant. Indeed, the HC extraction from graphene-2D PV device was demonstrated for the first time using TiO_*x*_–Ti heterostructures where an open circuit voltage up to 0.3 V was reached, 2 orders of magnitude higher than the devices relying on thermoelectric effects [125]. Graphene can also serve as an excellent HC absorber and thus be a promising candidate for realizing efficient HC solar cells. The power conversion efficiency is capped to 33% under 1 sun AM1.5 for conventional p–n junction solar cell with an optimum bandgap [126], and quite remarkably for an ideal hot carrier solar cell (that utilizes the total free energy of the absorbed photons without thermal loss), it has been calculated to increase up to 66% at the highest achievable electronic temperature of ∼3000 K [127]. In particular, the internal quantum efficiency for a graphene–WS_2_ heterostructure is reported to be as high as 55% with 1.6 eV photoexcitation [128]. We refer the interested reader to a recent review on this subject for more detail [70].

#### 4.2.2 Hydrovoltaic energy generators and ion pumps

Water contains tremendous energy in various forms and captures a remarkable proportion of the total solar energy received by Earth [129]. Therefore, strategies to extract and convert this energy have a great potential to provide new pathways for sustainable energy generation and storage. For example, extraction of energy from salinity gradient (*blue energy*) or its reverse process (*ion pumps*) has recently attracted great interest [130]. Furthermore, nanostructured materials can generate electricity on interaction with water and its dissolved ions via different electrokinetic effects, a process that is now referred to as *hydrovoltaic* energy conversion [131], [132], [133]. Due to their low-dimensionality and highly accessible π-bonds, carbon-based nanomaterials have shown attractive characteristics for these latter applications. Also, unique physical interactions have been reported. For example, it was proposed and later on shown that water flow along a CNT can excite a phonon wind that drags the free carriers, creating up to 1 mA of current and several millivolts of voltage [134], [135], [136].

Graphene, together with its doped forms, stands out among the carbon-based nanomaterials. In fact, high carrier mobility plays an important role in enhancing the net electrical power generated during the solid–ion interaction. Additionally, graphene out-plane π-bonds can more easily interact with external charged species (ions, molecules, etc.) and physical fields [137]. Most importantly, the possibility to modulate graphene electronic properties provides additional degrees of freedom in controlling this unique solid–liquid interaction. So far, the vast majority of works have leveraged chemical functionalization of graphene, i.e., grafting of relevant chemical groups to enhance its interaction with external ionic species [138, 139]. For example, graphene oxide (GO) layers have shown remarkable ion pumping properties under asymmetric illumination. This has been attributed to the different diffusivities and mobilities of electrons and holes in GO resulting in charge redistribution and generation of a net voltage capable of driving ion transport against the concentration gradient [130, 140, 141]. Interestingly, because of their functionalization, graphene oxide (GO) sheets are cation selective and there is a positive correlation between photocurrent and surface charge density. It is imperative to note that pristine graphene is not an ideal material as an active surface for ion pumps based on the above mechanism, primarily due to its lack of ion-selectivity and similar electron–hole mobilities. Recently, a plasmonic enhanced approach has been utilised to increase light absorption and improve photo-excited charge carrier diffusion [142]. Indeed, GO decorated with Ag nanoparticles was capable of transporting ions uphill a 40-fold concentration gradient.

Photoinduced electrostatic gating of graphene, however, can also offer interesting modulation of these phenomena. Indeed, the hydrophilicity of graphene, which is a measure of solid–liquid interaction, is connected to the position of its fermi level [143, 144]. Additionally, water flow over graphene has been recently shown to directly tune its fermi level [145]. Specifically, Yin et al. reported that motion of a liquid drop can generate voltage of several millivolts by driving the charging and discharging of the pseudocapacitance formed at the solid–liquid interface [133]. Importantly, the generated currents and voltages are directly related to the conductivity of the graphene layer. Thus, the performance of the device could be enhanced by modulating the fermi-level of graphene through illumination [12] (Figure 8c). Lin et al. have extended the functionality of such a graphene hydrovoltaic generator by leveraging the complementary availability of water and sunlight to generate a voltage of several millivolts [12] (Figure 8d). It is worth mentioning that the energy generation based on the coupling of ionic and electronic subsystems lacks the microscopic interpretation especially when fluid flow is continuous rather than isolated droplets. For the latter case it is reported that current is generated as a result of doping and dedoping of the semiconductor induced by the motion of the droplet [146].

### 4.3 Data processing

**Figure 9:**
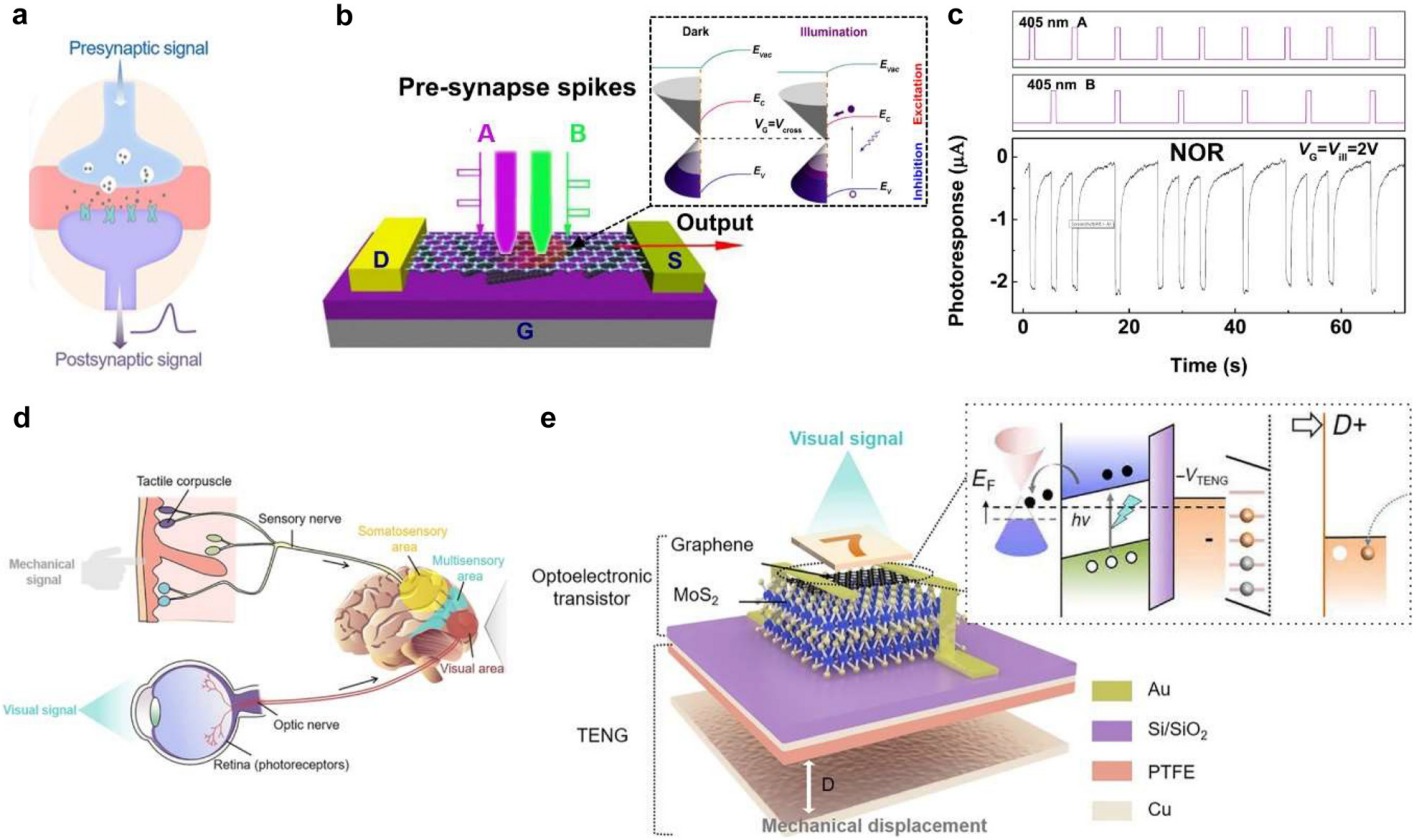
Data processing applications of graphene. (a) Schematic of biological synaptic transmission. (b) Artificial axon-multi-synaptic network based on graphene-SWCNTs hybrids under multiple light spikes. Inset image is the band diagram of graphene/SWCNTs interface before and after illumination. (c) The dynamic photoresponse of the NOR logic operation of the network. (d) Schematic illustrations of biological tactile/visual sensory systems. (e) Schematic structure of a graphene/MoS_2_ heterostructure-based artificial synapse. The inset is the band diagram of the graphene/MoS_2_ mechano-optoelectronic transistor at separation state. Panel (b) and (c) are adapted with permission from [152], Panel (a), (d) and (e) are adapted with permission from [13].

Photonic neuromorphic computing is an emerging field of research, which mimics the complex neural network (consisting of neurons and synapses) of the human brain. It can efficiently deal with highly sophisticated tasks with high speed and low energy consumption. It is superior to that of traditional supercomputers based on separate core memory and processing units. Figure 9 shows the signal processing in neurons. By integrating inputs from other neurons, they generated spikes as a threshold is reached. Synapses connect presynaptic neurons and postsynaptic neurons, and carry out the computation according to neuronal activities [147]. In analogy to this functioning mechanism, a photonic memristor has a tunable conductance, equivalent to a synaptic weight, while photon stimulation or electrical stimulation is regarded as a synaptic spike.

The current research on photonic neuromorphic devices mainly includes: chalcogenide phase change materials (PCM) devices [148], long synapses element based on Ge_2_Sb_2_Te_5_ islands deposited on top of Si_3_N_4_/SiO_2_ waveguides [149], and ultra-compact hybrid Ge-Sb-Se-Te-Si Mach–Zehnder modulator with electrothermal switching mechanism [150]. These devices can achieve all-optical fast zero-power nonlinear response with long-term information retention capability. To further increase the high integration of the system, 2D materials have also been extensively studied for photonic memory. Recently, because of their broadband spectral region, low power consumption and intrinsic optoelectronic properties, lots of nanomaterials have been employed to fabricate optoelectronic synaptic device such as silicon nanocrystals [122], graphene [123], CNTs [124], metal sulphides [125], and perovskite [126]. Due to the outstanding optical modulation of graphene, there have been many reports of its application in photonic memory and photonic synapses using a field-effect transistor approach [151, 152]. For these devices, optical signals and gate bias play important roles in programming/erase operations, and source-drain voltage is used for read-out current. Currently, photonic memory based on graphene are mostly three-terminal lateral photonic devices, which take advantage of the photo-induced charge trapping/detrapping on the floating gate. The conductance of the graphene-based nanochannel is employed to encode synaptic weights [152]. Importantly, in order to amplify the light-input, most graphene-based optoelectronic synapses are hybrids that incorporate nanoparticles into graphene, or VdWs heterostructures. Pradhan et al. [153] developed ultrathin photonic synapses using perovskite quantum dots grown directly on the graphene lattice which showed excellent light-assisted memory effect, such as short-term plasticity, long-term plasticity, and long-term depression. The low energy consumption of 36.75 pJ per spike enables the application of neuromorphic architecture to mimic the human brain functionalities. As shown in Figure 9b, Qin et al. [152] reported a light-activated synaptic device based on a graphene-SWCNTs phototransistor. Since the photogenerated holes in the hybrids are partially captured in the trap sites (such as intrinsic defects, dangling bonds, and interfaces of heterostructures), the devices can maintain the stable shutter effect for a long time even after the incident light is switched off because of the high trapping energy barrier. To demonstrate the optical computing capabilities, they applied two laser pulses (405 nm A and B light pulses with different periods, defined as pre-synapse A and pre-synapse B) as stimuli to this synaptic device. As shown in Figure 9c, individual pre-synapse stimulation or both pre-synapses triggered simultaneously could induce negative output current, which worked as NOR logic operations. Moreover, they also demonstrated AND and OR logic operations based on this device through coupling with external light fields. It provides an important basis for complex photonics logic operations and enables integrated photonic neuromorphic computing architecture.

Furthermore, these device-level photonic synapses have been used to construct large-scale crossbar arrays which form photonic artificial neural networks. These artificial neural networks validate efficient neuromorphic calculations to achieve artificial intelligence functions, such as pattern recognition and image processing. Yu et al. [13] proposed a bioinspired mechano-photonic artificial synapse which is composed of a graphene/MoS_2_ heterostructure-based phototransistor and an integrated triboelectric nanogenerator (TENG) (see Figure 9d and e). The optoelectronic synaptic behaviors, e.g., postsynaptic photocurrents, persistent photoconductivity, and photosensitivity of graphene/MoS_2_ transistor, can be modulated by the triboelectric potential induced by TENG displacement because the triboelectric potential can efficiently control the charge transfer/exchange between graphene and MoS_2_. They further proved that the artificial neuromorphic visual system based on an artificial neural network formed by this artificial synapse can significantly improve the image recognition accuracy to 92%.

### 4.4 Light modulation

Ultimately, graphene-based systems can be used to control light with light. In particular, in the following we discuss its use for controlling light emission addressing both optical pulse generation and thermal radiation emission.

#### 4.4.1 Optical pulse generation

**Figure 10:**
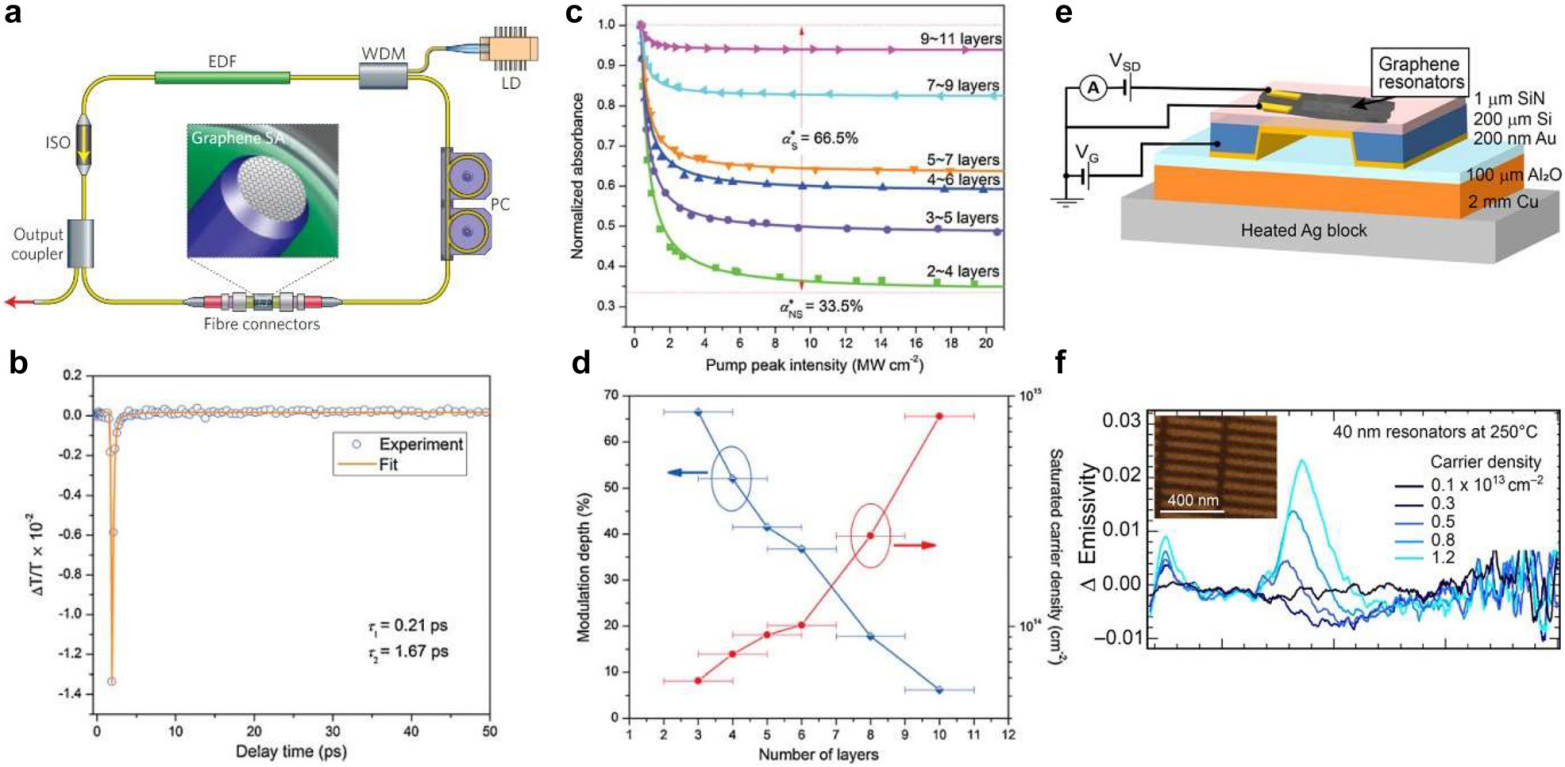
Light-modulation applications of graphene. (a) Graphene mode-locked ultrafast laser: a graphene SA is inserted between two fiber connectors. An erbium-doped fiber (EDF) is the gain medium, pumped by a laser diode (LD) with a wavelength-division multiplexer (WDM). An isolator (ISO) maintains unidirectional operation, and a polarization controller (PC) optimizes mode-locking. (b) Measured transmissivity transients for multilayer graphene. The open circles are the experimental data and the solid curve is analytical fit to the data using exponentials with time constants *t*_1_ and *t*_2_. The fast relaxation time *t*_1_ corresponds to carrier–carrier intraband scattering rates and the slow relaxation time *t*_2_ correlates with electron–hole interband recombination. (c) Nonlinear absorption of graphene films vs excitation intensity for different numbers of graphene layers. The dots are the experimental data and the solid curves are analytical fits to the data using Eq. (4). (d) Modulation depth and saturated carrier density versus number of graphene layers. (e) Graphene resonators array placed on the temperature-controlled stage with electrostatic gating for emissivity measurements. (f) Carrier density dependence of change in emissivity with respect to the charge neutral point (CNP) for 40 nm graphene nanoresonators (inset) at 250 °C. Panel (a) is adapted with permission from [7]; Panel (b)–(d) are adapted with permission from [156]. Panel (e) and (f) are adapted with permission from [160].

In most ultrafast lasers, a critical optical element is the SA which converts the continuous wave into an ultrafast laser pulse (Figure 10a). For a material to be used as SA, it needs to be highly optically nonlinear and have low optical loss. Current state-of-the-art is a semiconductor SA mirror. Other materials are also widely explored such as CNTs, graphene, TMDs, and black phosphorus [154, 155]. As we discussed in Section 2, graphene has a broadband and frequency-independent absorption that, combined with Pauli blocking, ensures that the interband optical transitions in zero-band graphene can be readily saturated under strong excitation. Thus, combined with ultrafast carrier dynamics, graphene’s nonlinear optical properties can be leveraged for the realization of an SA for ultrafast pulse generation. Bao et al. demonstrated for the first time that graphene can be used as an SA in a mode-locked fiber laser for ultrashort (756 fs) pulse generation with a tunable modulation depth by varying the number of graphene layers [156] (Figure 10b–d). Moreover, the saturation intensity is tunable with the number of graphene layers, and the modulation depth is reduced from 66.5 to 6.2% when the number of graphene layers is varied from 3 ± 1 to 10 ± 1, which is due to the increased nonsaturable loss caused by enhanced interlayer scattering. The saturated carrier density for 9–11 layers of graphene is 3 orders of magnitude higher than that of traditional semiconductor SA, which qualifies graphene as the potential material for the generation of low-noise laser pulses.

The optical nonlinearity becomes more favorable for ultrafast laser applications when several layers of graphene are stacked together giving rise to more density of states in the band structure. Therefore, yet another stable mode-lock laser was investigated using multilayered graphene which leads to significant reduction in modulation depth up to 2.93% and much shorter pulse width of 432.47 fs and 6.16 nm bandwidth [157]. The primary function of graphene as an SA is to initiate and sustain pulsing operation against continuous wave operation that can be achieved either by mode locking or Q-switching. While previous reports used mode-lock operation in the 1–1.5 μm spectral region, Tang et al. fabricated an SA mirror working at 2 μm (important for applications in lidar, molecule spectroscopy, etc.) wavelength and 6.1 nm bandwidth demonstrating both mode-locking and Q-switching [158]. Another possibility is to use graphene as a common SA for syncing two mode-locked lasers working at different wavelength and pulse widths of 915 fs and 1.57 ps [159]. The authors investigated the synchronization mechanisms and found that the nonlinear interaction of the generated pulses was enhanced in the presence of a common graphene SA. Thus, the use of graphene in ultrafast lasers has been steadily increasing as it opens new possibilities and also because it is simple and cost-effective fabrication and integration. However, specific requirements have to be met before using it commercially, such as engineering the key parameters like modulation depth, saturation intensity and nonsaturable loss. On the other hand, the extensive use of semiconductor SA mirror in this context is mainly credited to the well-developed semiconductor technologies which allows for example band and defect engineering and synthesis that provides effective control over the key parameters mentioned above.

#### 4.4.2 Modulation of thermal emission

Thermal emission modulation by heating and cooling of macroscopic objects is slow and inefficient. Indeed, the typically large heat capacity of the object requires transferring of a large amount of energy, which takes a long time. An alternative way to modulate thermal emission is to control the emissivity of the object. Kirchhoff’s law states that the angular spectral emissivity is equal to the angular spectral absorptivity for the reciprocal thermal emitters (satisfying the Lorentz reciprocity). Thus, photonic devices that are able to dynamically modulate the absorption of incident light can also actively modulate their own thermal emission. The direction, polarization, and the spectral line shape of thermal emission can also be controlled by engineering the absorption characteristics of the emitter [161]. Active manipulation of emissivity becomes possible in electrically tunable metasurfaces [162], [163], [164] and metamaterials [165, 166] where light–matter interaction can be manipulated at subwavelength scale. In particular, plasmonic metasurfaces provide a strong far-field coupling, phase tunability, and polarization selectivity, and thus, are well fit for the role of tunable thermal emitters [164].

Graphene nanostructures with electrically tunable plasmon resonances provide a platform for the dynamic thermal emission modulation. Brar et al. showed that the thermal emission of an array of plasmonic nanoresonators patterned in graphene on SiN_*x*_/Au substrate can be dynamically tuned via modulation of the graphene Fermi level [160] (Figure 10e and f). Change of the emissivity spectra peaks at the plasmon resonance frequency. As the carrier density in graphene varies from 0.5 to 1.2 × 10^12^ cm^−2^ (by the electrostatic doping), the intensity of the peak substantially increases and its spectral position blue shifts. Since the peak originates from the graphene plasmon resonance, the emissivity spectrum is also strongly dependent on the polarization. As predicted by the Kirchhoff’s law of thermal radiation, the emissivity and the absorptivity spectra of the device at the same carrier density match well. The maximum thermal power modulation is observed to be 50 pW cm^−1^ at 250 °C which is comparable with commercial mid-IR LEDs. Because graphene is a 2D material with a very small heat capacity, the switching speed of the thermal emission modulation is limited only by the RC time constant rather than the heat capacity or thermal time constant of the device [160]. As a final remark, we note that there exist interesting possibilities to control thermal emission using light illumination by employing previously described photo-modulation mechanisms such as the PTO effect and the photo-doping at graphene heterojunctions. In principle, there should be no difference between the electrically- and optically-driven thermal emission modulation provided that the induced change of carrier concentration (i.e., the graphene plasmonic response) is the same.

## 5 Conclusions and perspective

In conclusion, it is clear that photo-modulation of graphene optical and electrical properties offers unprecedented opportunities for device applications across many areas. Yet, fulfilling this potential will require further advances in fundamental understanding, material engineering and device architecture.

In particular, it is critical to improve our comprehension of hot carrier dynamics and transport as well as their coupling with other excitations (plasmons, excitons). For example, it has been recently shown that coupling between graphene and MoS_2_ significantly slows down the thermalization dynamics of photoexcited carriers in graphene [167]. While this effect is extremely beneficial for energy conversion devices, as it enhances hot carrier collection probability, it could be detrimental for light-modulation applications where a fast dynamic is necessary. Also, theoretical studies have previously suggested a unique ultrafast radiative heat transfer regime between plasmon-supporting graphene nanostructures [168]. Although cooling of hot carriers via plasmon emission has been recently observed experimentally [37], direct applications to radiative cooling have not been addressed yet. Similarly, despite its potential for applications in ultrafast optical sensing in the infrared regime, the recently proposed PTO effect has not been demonstrated experimentally [53]. Yet, recent reports of highly efficient THz high-harmonic generation [58] and highly sensitive microwave bolometry [59] do confirm the broad applicability of this effect for light detection and emission devices. On the other hand, PTE devices have been recently reported having operational bandwidth approaching 100 GHz [50, 63], which is in the same order of magnitude as the theoretically predicted limit of 500 GHz corresponding to the lifetime of hot carriers [169]. Finally, understanding the impact of parasitic thermal effects during optical excitation will be critical to establish theoretical performance limits of optically modulated graphene-based devices. Overall, achieving greater control over nonequilibrium, ultrafast phenomena in graphene will be critical to exceed the thermodynamic limit of photovoltaic devices, achieve the highest modulation speeds for photodetection and data processing devices and ultimately enable new applications (i.e., sensing and spectroscopy).

Concurrently, material-related improvements have an important role to play in overcoming specific limitations of graphene while retaining its unique modulation capabilities. For example, twisted bilayer graphene has already demonstrated the emergence of a whole new class of physical properties, including superconductivity [170]. Also, chemically functionalized graphene oxide has shown better performances for hydrovoltaic energy conversion devices, although optimization of the functionalization process and precise analysis of the role of the different functional groups have received little attention. Finally, engineering of 2D heterostructures has opened almost unlimited opportunities for tailoring their optoelectronic properties while retaining excellent photo-modulation capabilities across a very broad spectral range. Combined with fast progress in large-area synthesis of vast classes of 2D materials (e.g., TMDs), these heterostructures will eventually be able to go beyond the laboratory scale to enter real world devices.

To conclude, graphene-based photomodulated devices have made huge advancements and have reached a high level of functionality, as demonstrated by the discussed neuromorphic device transducing optical inputs into mechanical signals. Therefore, by addressing pending physical and material science questions they are expected to play a critical role in a growing number of application domains.
